# V2a Neurons Constrain Extradiaphragmatic Respiratory Muscle Activity at Rest

**DOI:** 10.1523/ENEURO.0492-18.2019

**Published:** 2019-08-05

**Authors:** Victoria N. Jensen, Kari Seedle, Sarah M. Turner, John N. Lorenz, Steven A. Crone

**Affiliations:** 1 Neuroscience Graduate Program, University of Cincinnati College of Medicine, Cincinnati, OH 45219; 2 Division of Neurosurgery, Cincinnati Children’s Hospital Medical Center, Cincinnati, OH 45229; 3 Department of Pharmacology and Systems Physiology, University of Cincinnati College of Medicine, Cincinnati, OH 45267; 4 Division of Developmental Biology, Cincinnati Children’s Hospital Medical Center, Cincinnati, OH 45229; 5 Department of Neurosurgery, University of Cincinnati, College of Medicine, Cincinnati, OH 45267

**Keywords:** accessory respiratory muscle, diaphragm, electromyography, interneuron, respiration, V2a neuron

## Abstract

Breathing requires precise control of respiratory muscles to ensure adequate ventilation. Neurons within discrete regions of the brainstem produce oscillatory activity to control the frequency of breathing. Less is understood about how spinal and pontomedullary networks modulate the activity of respiratory motor neurons to produce different patterns of activity during different behaviors (i.e., during exercise, coughing, swallowing, vocalizing, or at rest) or following disease or injury. Here, we use a chemogenetic approach to inhibit the activity of glutamatergic V2a neurons in the brainstem and spinal cord of neonatal and adult mice to assess their potential roles in respiratory rhythm generation and patterning respiratory muscle activity. Using whole-body plethysmography (WBP), we show that V2a neuron function is required in neonatal mice to maintain the frequency and regularity of respiratory rhythm. However, silencing V2a neurons in adult mice increases respiratory frequency and ventilation, without affecting regularity. Thus, the excitatory drive provided by V2a neurons is less critical for respiratory rhythm generation in adult compared to neonatal mice. In addition, we used simultaneous EMG recordings of the diaphragm and extradiaphragmatic respiratory muscles in conscious adult mice to examine the role of V2a neurons in patterning respiratory muscle activity. We find that silencing V2a neurons activates extradiaphragmatic respiratory muscles at rest, when they are normally inactive, with little impact on diaphragm activity. Thus, our results indicate that V2a neurons participate in a circuit that serves to constrain the activity of extradiaphragmatic respiratory muscles so that they are active only when needed.

## Significance Statement

Extradiaphragmatic respiratory muscles are crucial for increasing ventilation during exercise or following disease and injury, yet the neural mechanisms that ensure that they are recruited only when needed are not known. By silencing the activity of V2a neurons we reveal an important role for these neurons in preventing extradiaphragmatic respiratory muscle activity at rest. Unlike in neonatal mice, silencing V2a neurons in adult mice does not disrupt respiratory rhythm generation or have a major impact on diaphragm activity. Our results support the feasibility of targeting V2a containing circuits to control extradiaphragmatic muscles without impairing respiratory rhythm generation to treat patients with neuromuscular disease or spinal cord injury.

## Introduction

A major challenge in the study of respiratory control is to understand how different neurons modulate respiratory motor output to maintain ventilation during different behaviors, stages of development, or following disease and injury. For example, healthy mammals at rest rely predominantly on the diaphragm for inspiration. When ventilatory demands increase (i.e., during exercise), there is an increase in the activity of extradiaphragmatic respiratory muscles that include inspiratory pump muscles (e.g., external intercostal and scalenes) as well as accessory respiratory muscles (e.g., trapezius, pectoralis, and sternocleidomastoid; Sieck and Gransee, 2012) that we will refer to collectively as auxiliary respiratory muscles (ARMs). ARMs are also recruited in patients with neuromuscular disease, spinal cord injury, or diaphragm paralysis to compensate for impaired diaphragm function (Bennett et al., 2004; Sieck and Gransee, 2012; Johnson and Mitchell, 2013). However, it is not known why some patients with diaphragm impairment use ARMs more than others, as the neural mechanisms leading to recruitment of ARMs during exercise or following disease and injury are poorly understood. A better understanding of circuits patterning respiratory muscle activity would facilitate the development of therapies to improve ventilation, reduce respiratory infections, and prevent ventilation failure in patients with neuromuscular disease and injury.

The present study investigates the roles of V2a neurons, a class of glutamatergic neurons in the brainstem and spinal cord marked by Chx10 expression, in the control of respiratory rhythm and pattern in adult mice. A prior study showed that ablating V2a neurons during development results in slow and irregular breathing in neonates, likely due to a loss of excitatory drive from V2a neurons in the medial reticular formation to neurons in the pre-Bötzinger complex (Crone et al., 2012). However, these experiments could not conclusively distinguish whether the changes in breathing were the direct result of loss of V2a neuron function versus developmental defects resulting from the loss of V2a neurons in the embryo. In addition, a subset of these mice was able to survive past the neonatal period and their breathing pattern appears regular after the first week, indicating that V2a neurons might be dispensable for normal breathing in mature mice. Alternatively, ablation of V2a neurons in the embryo may lead to developmental compensation by other cell types to maintain ventilation in mature animals. Thus, it is not yet clear whether V2a neuron function is critical for a normal respiratory rhythm in mature animals.

In addition to modulating respiratory rhythm and frequency, previous studies suggest that V2a neurons may be important for patterning respiratory muscle activity. Cervical spinal V2a neurons are synaptically connected to phrenic motor neurons and thus could modulate diaphragm function (Zholudeva et al., 2017). The number of V2a neurons connected to phrenic motor neurons increases following spinal cord injury, indicating that V2a neurons may be particularly important for respiratory compensation following injury. In addition, it has been suggested that degeneration of V2a neurons in a mouse model of ALS could account for the failure of accessory respiratory muscles to be activated for breathing at late stages of disease (Romer et al., 2017). This hypothesis was based on the observation that increasing the excitability of V2a neurons using an excitatory designer receptors exclusively activated by designer drugs (DREADD) in healthy adult mice activates scalene and trapezius respiratory muscles at rest, when these muscles are mostly inactive (Romer et al., 2017). Thus, V2a neurons have the potential to influence the number and type of respiratory motor neurons recruited for breathing. However, it is not yet known whether V2a neural activity is required for patterning respiratory motor activity in adult mice.

Here, we target expression of an inhibitory DREADD (hM4Di or “(G_i_)DREADD”; Urban and Roth, 2015) to Chx10 expressing neurons to test whether normal activity of V2a neurons is required for a regular breathing rhythm and frequency in healthy neonatal and adult mice and to evaluate their role in patterning activity of respiratory muscles in adult mice. We find that the activity of V2a neurons is important for modulating the frequency and regularity of respiratory rhythm in neonatal mice, but only modulates the frequency of respiration in adult mice. In addition, we show that silencing V2a neurons results in activation of ARMs in adult mice at rest, suggesting that these neurons play an important role in patterning extradiaphragmatic respiratory muscle activity.

## Materials and Methods

### Animals

All animal procedures were performed according the National Institutes of Health guidelines and approved by the institution’s animal care committee’s regulations. V2a-(G_i_)DREADD mice were generated by breeding Chx10^Cre/+^ mice (Azim et al., 2014; Romer et al., 2017) to ROSA^PNP-(Gi)DREADD/+^ (B6N.129-Gt(ROSA)26Sortm1(CAG-CHRM4*, -mCitrine)Ute/JJax, stock #026219, The Jackson Laboratory) mice to generate Chx10^Cre/+^; ROSA^PNP-(Gi)DREADD/+^ mice. Adult male Chx10^Cre/+^ mice (lacking the ROSA^PNP-(Gi)DREADD/+^ allele) were used as non-DREADD expressing controls. On rare occasions, we observe recombination outside of the nervous system as a result of “leaky” germline or early embryo expression of Cre recombinase. Therefore, we perform PCR on DNA isolated from the tail of all animals to identify any with the recombined ROSA^P-(Gi)DREADD/+^ allele and these animals are excluded from all experiments and breeding (primers: 5'-CTCTG CTAAC CATGT TCATG C-3' and 5'-GAAGG CGCCT ATGAT GAGAT C-3'). Genotyping was performed by PCR using primers to detect the Chx10^Cre^ (5’-GCATT AGACA CCGGA GGG-3’ and 5’-GGACA GAAGC ATTTT CCAG-3’), Chx10 wild type (5’-GCATT AGACA CCGGA GGG-3’ and 5’-CTCCC GACTG TGACT TTCC-3’), ROSA^PNP-(Gi)DREADD^ (5'-ATGTC TGGAT CCCCA TCAAG-3' and 5'-GAAGG CGCCT ATGAT GAGAT C-3') and ROSA wild type (5'-AAGGG AGCTG CAGTG GAGTA-3' and 5'-GAAAA TCTGT GGGAA GTC-3') alleles.

### Histology, imaging, and quantification

Immunohistochemical and imaging analysis was performed on spinal cords and medulla of neonatal and adult V2a-(G_i_)DREADD mice. Specificity of the HA antibody was confirmed by lack of staining in Chx10^Cre/+^ and ROSA^PNP-CHRM4/+^ mice that lack expression of the HA-tagged (G_i_)DREADD. Neonatal mice were placed on ice for 4 min and transcardially perfused with 4% paraformaldehyde (PFA) in phosphate buffer (PB; 770 mM Na_2_HPO_4_⋅7H_2_O, and 231 mM NaH_2_PO_4_⋅H_2_O, pH 7.4). Brains and spinal cords were dissected and submerged in 4% PFA until 2 h after the start time of perfusion. Tissue was then rinsed in phosphate PBS (137 mM NaCl, 2.7 mM KCl, 10 mM Na_2_HPO_4_, and 2 mM KH_2_PO_4_, pH 7.2) overnight to remove PFA, cryoprotected in 30% sucrose overnight (all at 4°C), mounted in OCT medium and stored at –80°C. Transverse sections of cervical spinal cord and medulla were cut (14 μm) on a cryostat microtome and collected directly onto slides. Immunohistochemistry was performed as described previously (Thaler et al., 1999; Crone et al., 2009) using the following primary antibodies (and DAPI nuclear stain): Chx10 (guinea pig at 1:10,000), and HA-Tag C29F4 (rabbit at 1:1000, Cell Signaling #3724). Images were obtained on a Nikon A1 Plus inverted confocal microscope. The number of HA-Tag^+^, Chx10^+^, and HA-Tag^+^/Chx10^+^ cells with DAPI^+^ nuclei were counted from single plane confocal 20× images using NIS Elements Software. Every forth section at C4 (16–18 hemisections/animal) was counted for cervical sections and every forth section at the level of the nucleus ambiguous for medulla sections (18–20 hemisections/animal). The average number of cells per hemisection was calculated for each animal. C4 was used as a standard reference point based on the distinct morphology of the phrenic nucleus in this segment to ensure counts were made at a consistent axial level. Similarly, the distinct morphology of the nucleus ambiguous was used as a reference in the medulla.

Adult mice were anesthetized with pentobarbital (0.1 mg/g, i.p.) and were transcardially perfused with PB followed by 4% PFA in PB. Tissue was rinsed in PBS overnight, cryoprotected in 30% sucrose overnight (all at 4°C), mounted in OCT medium and stored at –80°C. Transverse sections of adult cervical spinal cords were cut (14 μm) on a cryostat microtome and collected directly onto slides. Transverse sections of adult medulla were cut (50 μm) on a cryostat microtome and collected as floating sections in PBS + 30% v/v ethylene glycol + 25% w/v glycerol. Immunohistochemistry was performed on adult tissue as described previously (Romer et al., 2017) using the following primary antibodies (and DAPI nuclear stain): Chx10 (sheep at 1:2000, Abcam #ab16141), and HA-Tag C29F4 (rabbit at 1:1000, Cell Signaling #3724). The number of HA-Tag^+^ cells, Chx10^+^ cells, and HA-Tag^+^/Chx10^+^ cells with DAPI^+^ nuclei were counted as described above in every eighth section at C4 (9–14 hemisections/animal) and in every third section at the level of the nucleus ambiguous (12–13 hemisections/animal). The average number of cells per hemisection was calculated for each animal.

### Surgical implantation of telemetry devices

Telemetry devices [either single (TAE-F10) or double channel (F20-EET) transmitters from Data Sciences International] were implanted as early as eight weeks of age to chronically record EMG from respiratory muscles of V2a-(G_i_)DREADD and non-DREADD control mice. Surgeries to implant devices subcutaneously for recording EMG from the trapezius and scalene muscles were performed as previously described (Jensen et al., 2017; Romer et al., 2017). Briefly, a 3-cm-long incision was made between the right shoulder and ear and the transmitter was placed subcutaneously on the back of the mouse. Two sets of biopotential leads were inserted ∼1 mm apart from one another into the trapezius and scalene muscles. The incision was closed and mice were allowed to recover for one week before recording.

Surgeries to implant devices intraperitoneally to record EMG from diaphragm and/or trapezius muscles were performed as follows. Mice were placed supine under isoflurane anesthesia. A “T” incision was made in the skin by making a 4-cm-long lateral incision directly below the xyphoid process and an adjoining 2-cm-long vertical incision was made from the middle of the lateral incision to the middle of the rib cage. Next, a 3-cm-long lateral incision was made through the abdomen muscle just below the rib cage. The xyphoid process was clamped with Hemostats to expose the diaphragm. The transmitter was placed on the left side of the abdominal cavity next to the intestines. One set of leads was set aside for later use (trapezius insertion) while the other set was positioned near the diaphragm. Forceps were used to gently lift up on the right side of the costal diaphragm. Forceps were used to insert the exposed wire of one biopotential lead through the first muscle layer of the diaphragm muscle without puncturing the second muscle layer. Extreme care was taken to ensure that the diaphragm was not punctured to prevent a fatal injury. The exposed wire was secured in place against the diaphragm muscle with a small drop of cyanoacrylate adhesive. A second biopotential lead was inserted in the same manner ∼1 mm caudal to the position of the first lead. Excess lead length was coiled and placed just below the abdominal muscle and above the implanted transmitter. The abdominal muscle was closed over the transmitter and excess coiled leads with suture, with care taken to externalize the second pair of biopotential leads for trapezius insertion. The skin incision was closed using inside out sutures, leaving ∼1 mm open closest to the mouse’ head where the second set of leads remained exposed. A second incision was made between the mouse’ right shoulder and ear to expose the trapezius muscle. A trocar was used to tunnel the second set of exposed leads subcutaneously from the intraperitoneally implanted transmitter to the open incision near the mouse’ shoulder. The leads were inserted into the trapezius muscle as described above. Mice were allowed to recover for one to two weeks before EMG data acquisition.

### Neonatal whole-body plethysmography (WBP)

WBP was performed on age P2 non-DREADD expressing mice and V2a-(Gi)DREADD mice. The barometric chambers were standardized by injecting 10 consecutive 10-μl pumps of air into an open port on the experimental chamber using a 100-μl Hamilton syringe. Animals were placed in a barometric chamber and equilibrated with open air flow for 5 min. The pressure difference between the sealed experimental and reference chambers was measured with a differential pressure transducer for two 60s recording sessions: one baseline session and one session 20 min after an intraperitoneal injection of 10 μl of 10.0 mg/kg bw clozapine-N-oxide (CNO). Data are expressed in relative units (R.U.) normalized to the average maximum value obtained during standardization with 10-μl pumps of air from a syringe. CNO solutions were made by dissolving CNO directly into saline. At least 10 min of open-air flow was allowed between trials. Signals were amplified, digitized, and low pass filtered (50 Hz). Data were collected and analyzed using LabChart 7.0 software (AD Instruments).

### Neonatal plethsymography analysis

LabChart 7.0 software (AD Instruments) was used to mark individual resting breaths to be analyzed for breathing frequency. At least 70 resting breaths were analyzed before and after CNO administration. An irregularity score (IS = ABS[(T_n_ – T_(n–1)_)/T_(n–1)_]*100) was calculated for each breath (Viemari et al., 2011; Sheikhbahaei et al., 2017). The average IS of at least 50 consecutive breaths for each animal under each condition (without and with CNO) is reported. Breaths during movement were excluded from analysis.

### Adult WBP and EMG acquisition in conscious animals

Plethysmography, EMG acquisition, and digital video recording was performed as described previously (Jensen et al., 2017; Romer et al., 2017) using DSI Ponemah Physiology Platform Acquisition software v.5.20. Briefly, mice were placed in a plethysmography chamber on top of a transmitter receiving pad and allowed to acclimate for 30 min. The transmitter was turned on by placing a strong magnet near the animal inside the chamber before data collection. A vehicle control (0.1 ml of saline) was injected IP. Mice were placed in their home cage for 2 min immediately following all injections before being placed into the plethysmography chamber to prevent association of a painful injection with the chamber. After 2 min of reacclimating to the chamber, the transmitter was turned on and at least 20 min of resting data were collected (characterized by mouse inactivity and regular breathing). Next, mice were injected intraperitoneally with either 1.0, 5.0, 10.0, or 15.0 mg/kg bw CNO, placed in their home cage for 2 min, and then reacclimated to the chamber for 2 min before data acquisition began for 1 h. The same animals were tested with multiple CNO doses, with at least 2 d between each dose to avoid desensitization of DREADD receptors. A vehicle control recording was performed before each dose of CNO. Only periods in which the animal is at rest (not moving in video recordings) are used for analysis.

### Adult plethysmography analysis

The Ponemah Physiology Platform Analysis software v.5.20 was used to mark individual resting breaths to be analyzed for breathing frequency (f), tidal volume (V_T_), minute volume (MV), peak inspiratory flow (PIF), inspiratory time (T_i_), expiratory time (T_e_), and total breathing cycle time (T_t_) as described previously (Jensen et al., 2017; Romer et al., 2017). V_T_/T_i_, a measure of respiratory drive, was calculated from these derived values. Periods of ARM activity (trapezius bouts) were identified as described above. Interbout intervals used for analysis excluded all bouts as well as the four breaths immediately preceding or following a bout. At least 100 resting breaths were analyzed for each vehicle control and CNO treatment period. The average IS of 100 consecutive breaths (the same breaths analyzed for ventilation parameters) for each animal under each condition (vehicle or CNO) is reported.

### EMG analysis in conscious animals

The raw EMG signal was high pass filtered at 1.5 Hz and the root mean square (RMS) was calculated using a 30-ms window. The frequency of bouts of trapezius or scalene activity was counted as described previously (Jensen et al., 2017). Briefly, ARM EMG activity is scored as a “bout” if it satisfies the following three criteria: (1) the RMS EMG trace must exhibit at least three consecutive points that are at least 50% higher than the surrounding baseline (each point represents a 30-ms window), (2) EMG activity occurs during resting breaths (i.e., sighs and movement artifacts are excluded), and (3) EMG activity occurs when the mouse is still (i.e., no movement or changes in posture) based on the synchronized video recordings. ARM bout frequency was calculated by dividing the total number of bouts observed by the total time analyzed. Interbout intervals used for analysis excluded all bouts as well as the four breaths immediately preceding or following a bout. For analysis of diaphragm EMG, the RMS was used to measure the peak amplitude, duration, and instantaneous frequency of each breath (burst of activity). Diaphragm EMG peak amplitude was normalized to the maximum ventilatory effort reached during sighs to reduce intra-animal variability (Mantilla, et al., 2011). At least 50 resting breaths were analyzed for each vehicle control and CNO treatment period.

### Diaphragm EMG acquisition in anesthetized animals

Terminal bilateral diaphragm EMG recordings were performed on adult V2a-(Gq)DREADD and V2a-(Gi)DREADD mice before and after CNO administration. Animals were anesthetized under isoflurane anesthesia and placed supine on a heating pad. A 4-cm lateral incision through the skin and abdominal muscle just below the xyphoid process exposed the intraperitoneal cavity and diaphragm. Bi-polar platinum electrodes (Grass Technology) connected to an amplifier (BMA-400 AC/DC Bioamplifier, CWE Inc.) were inserted into the diaphragm and secured with cyanoacrylate adhesive to record diaphragmatic activity via Spike2 Data Analysis software (Cambridge Electronic Design Limited). Electrodes were grounded with an additional lead inserted into the abdominal muscle. A 10-min baseline of bilateral diaphragm activity was recorded. CNO was then topically applied to the exposed intraperitoneal cavity and diaphragm EMG was recorded for 1 h.

### EMG analysis in anesthetized animals

The diaphragm EMG signal was digitally amplified (5000× gain) and band pass filtered (30–3000 Hz) with a sampling frequency of 6.25 kHz. EMG signals were further processed to remove DC noise and the RMS was calculated over a 30-ms window. ECG artifact was digitally filtered out using the ECGDelete02 Spike2 script (Cambridge Electronic Design). Diaphragm EMG peak amplitude was normalized to the maximum ventilatory effort reached during sighs to reduce intra-animal variability (Mantilla, et al., 2011). At least 50 bursts were analyzed for diaphragm EMG peak amplitude and regularity of bursting frequency for the baseline recording and following CNO treatment.

### Statistical analysis

Statistical tests from SigmaStat 13 (Systat Software, SPSS Inc.) were used to analyze the data (see Table 1). All data passed the Shapiro–Wilk test for normality, except as noted. A one-way repeated measures ANOVA (Holm–Sidak all pairwise comparisons *post hoc* analysis) was used to analyze differences within animals when comparing values from three or more conditions tested within the same animal (e.g., multiple doses of CNO on ARM recruitment). Paired *t* tests were used to analyze differences in treatments within the same animal where only two conditions existed (e.g., vehicle vs CNO). Student *t* tests were used to analyze differences between groups (e.g., V2a-(G_i_)DREADD vs Chx10^Cre/+^ mice). Non-parametric Wilcoxon signed-rank tests were performed on datasets that failed the test for normality (coefficient of variation of breathing frequency (CV_f_) and irregularity score (IS) between vehicle control and CNO-treated conditions). Statistical significance was set at alpha level 0.05, and values in figures are reported as mean ± SE.

## Results

### (G_i_)DREADD is expressed in V2a neurons in the spinal cord and brainstem of V2a-(G_i_)DREADD mice

**Figure 1.**
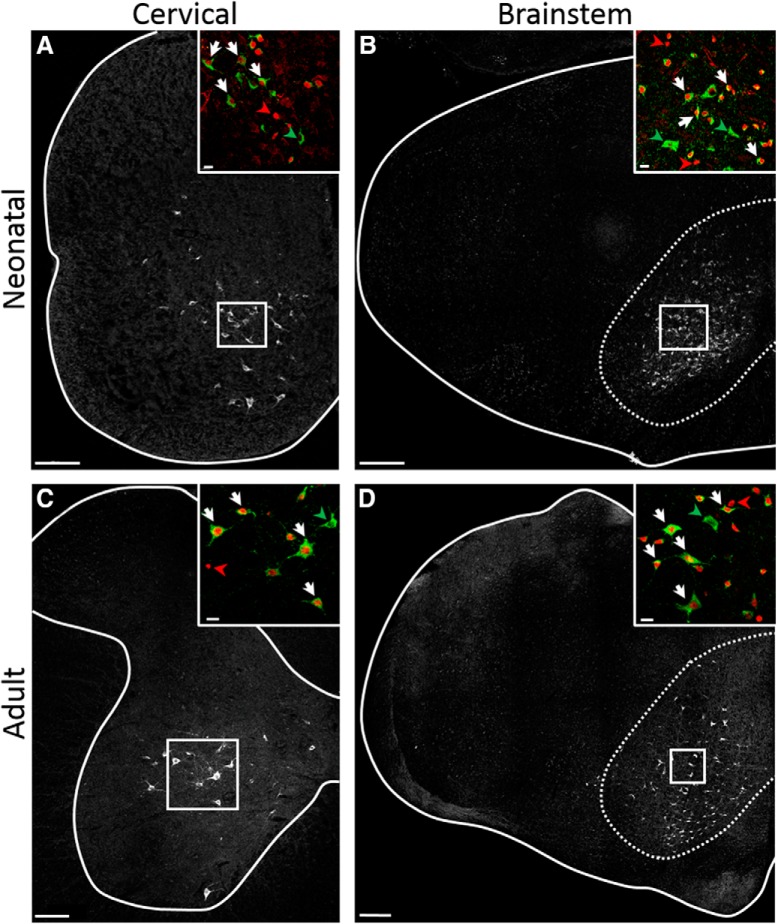
(G_i_)DREADD is expressed in V2a neurons in the spinal cord and brainstem of V2a-(G_i_)DREADD mice. Immunohistochemistry using antibodies to Chx10 and the HA tag on (G_i_)DREADD were used to label the nuclei of V2a neurons and (G_i_)DREADD expressing cells, respectively, in V2a-(G_i_)DREADD mice. ***A***, C4 cervical spinal cord section (spinal cord boundary outlined) of a neonatal (P2) mouse showing (G_i_)DREADD expression in the intermediate lamina where V2a neurons are found. Scale bar = 100 μm. ***B***, Coronal section of the medulla of a neonatal (P2) mouse at the level of the nucleus ambiguous showing (G_i_)DREADD expression in the medial reticular formation outlined (dotted line) where V2a neurons are found. Scale bar = 200 μm. ***C***, C4 cervical spinal cord section (gray matter outlined) of an adult mouse showing (G_i_)DREADD expression in the intermediate lamina where V2a neurons are found. Scale bar = 100 μm. ***D***, Coronal section of the medulla of an adult mouse at the level of the nucleus ambiguous showing (G_i_)DREADD expression in the medial reticular formation outlined (dotted line) where V2a neurons are found. Scale bar = 200 μm. ***Insets***, Boxed regions in ***A–D***. (G_i_)DREADD^+^ cells (green) predominantly co-label with Chx10^+^ nuclei (red; examples indicated by white arrows). Chx10^+^ neurons without (G_i_)DREADD expression (examples indicated by red arrowheads) and (G_i_)DREADD cells without detectable Chx10^+^ (examples indicated by green arrowheads) are infrequently observed. Scale bars = 20 μm.

To test the role of V2a neurons in modulating respiratory rhythm and motor pattern, we generated V2a-(G_i_)DREADD mice (Chx10^Cre/+^; ROSA^PNP-(Gi)DREADD/+^) to decrease the excitability of V2a neurons following acute injections of CNO (Urban and Roth, 2015; Zhu et al., 2016). Chx10^Cre/+^ mice, in which Cre recombinase has been inserted into the endogenous Chx10 locus by homologous recombination, have previously been used to reliably drive reporter gene expression in V2a neurons that express Chx10 during development or in the adult (Azim et al., 2014; Bouvier et al., 2015; Romer et al., 2017; Hayashi et al., 2018). Viruses expressing the (G_i_)DREADD receptor have previously been used to inhibit V2a neuron activity in spinal neurons to assess their role in fine motor control (Ueno et al., 2018), but breathing was not assessed in this study. We used antibodies to the hemagglutinin tag (HA-Tag) on the (G_i_)DREADD receptor and antibodies to Chx10 to mark the nuclei of V2a neurons to assess the distribution of (G_i_)DREADD in the cervical spinal cord (C4) and medulla of neonatal and adult V2a-(G_i_)DREADD mice (Fig. 1). The pattern of (G_i_)DREADD expression is similar in neonatal (postnatal day 2) and adult animals. HA-Tag^+^ cells have neuronal morphology and are located where V2a neurons are found: the intermediate laminae (but not dorsal horn) of the spinal cord and medial reticular formation of the medulla, consistent with prior studies using Chx10^Cre/+^ mice to selectively drive reporter expression in V2a neurons (Azim et al., 2014; Bouvier et al., 2015; Romer et al., 2017; Hayashi et al., 2018). We detected HA-Tag^+^ immunoreactivity in an average of 24.1 ± 0.6 (*n* = 5), 11.1 ± 0.4 (*n* = 6), 100.6 ± 10.9 (*n* = 3), and 112.9 ± 7.5 (*n* = 3) cells/hemisection in neonatal spinal cord, adult spinal cord, neonatal medulla, and adult medulla, respectively, of V2a-(G_i_)DREADD mice. This represents 77 ± 0.8% of Chx10^+^ neurons in neonatal and 80 ± 1.3% of Chx10^+^ neurons in adult cervical cord. In the medulla (at the level of the nucleus ambiguous) we detect HA-Tag^+^ immunoreactivity in 65 ± 4% of Chx10^+^ neurons in neonatal and 58 ± 3% of Chx10^+^ neurons in adult V2a-(G_i_)DREADD mice. HA-Tag^+^ cells are not observed in mice lacking the ROSA^PNP-(Gi)DREADD/+^ allele in P2 neonates (*n* = 5) or adults (*n* = 4), confirming the specificity of our antibody. As expected from prior studies showing that ∼50% of V2a neurons in the cervical cord that express Chx10 during embryonic development have low (detectable by RNA sequencing but undetected by antibodies) Chx10 expression after birth (Hayashi et al., 2018), we found that 47 ± 2% of HA-Tag^+^ neurons of P2 neonatal mice did not express detectable levels of Chx10. Similarly, 35 ± 6% of HA-Tag^+^ cells in neonatal medulla, 23.3 ± 3% in adult cervical spinal cord, and 23 ± 3% in adult medulla do not express detectable levels of Chx10. These results demonstrate that V2a-(G_i_)DREADD mice express the inhibitory DREADD receptor in the majority of V2a neurons and the extent and pattern of expression is similar in neonatal and adult mice.

### Decreasing the excitability of V2a neurons results in slow and irregular breathing in V2a-(G_i_)DREADD neonatal mice

**Figure 2.**
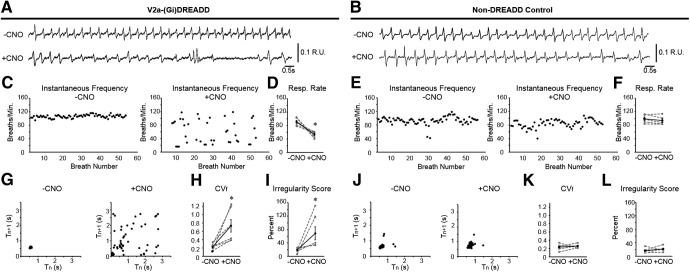
Decreasing the excitability of V2a neurons causes irregular breathing in neonatal V2a-(G_i_)DREADD mice. Representative traces of WBP before (top trace) and after (bottom trace) 10.0 mg/kg bw CNO treatment in a (***A***) V2a-G_i_(DREADD) mouse and a (***B***) non-DREADD control (Chx10^Cre/+^) mouse at P2. ***C***, ***E***, Instantaneous respiratory frequency is plotted for at least 50 consecutive breaths in V2a-(G_i_)DREADD mice and non-DREADD control mice before (left) and after (right) CNO treatment to illustrate the breath to breath consistency of respiratory frequency. ***D***, ***F***, Respiratory rate is decreased after silencing V2a neurons in V2a-(G_i_)DREADD mice but remains unchanged following CNO treatment in non-DREADD control mice. Poincare maps generated by plotting the respiratory cycle period (T_n_) versus the subsequent respiratory cycle period (T_n+1_) from the same trials shown in ***C***, ***E*** show an inconsistent period following CNO treatment in V2a-(G_i_)DREADD mouse (***G***) but not in the non-DREADD control (***J***). ***H***, CNO treatment (10.0 mg/kg bw) increases the CV_f_ compared to baseline in V2a-(G_i_)DREADD mice (*p* = 0.030, *n* = 6 but not in non-DREADD control mice (*p* = 0.619, *n* = 5; ***K***). ***I***, The average IS of the breathing frequency is increased in V2a-(G_i_)DREADD mice following CNO (10.0 mg/kg bw; *p* = 0.031, *n* = 6) but remains unchanged in non-DREADD control mice (*p* = 0.363, *n* = 5; ***L***). Gray dashed lines show individual animals. Solid black line shows the mean ± SE; *p < 0.05, paired *t* test or Wilcoxon signed-rank test (Fig. 1*I*).

Previous experiments in which V2a neurons were ablated during embryonic development could not conclusively distinguish if the changes in breathing were the direct result of loss of V2a neuron function versus developmental defects resulting from the loss of V2a neurons in the embryo. Therefore, we used whole body plethysmography (WBP) to measure respiratory frequency and regularity in postnatal day 2 V2a-(G_i_)DREADD mice before and after CNO treatment to determine the effects of acutely silencing V2a neurons on breathing (Fig. 2). The number of respiratory cycles/min was calculated before and after CNO treatment in both V2a-(G_i_)DREADD mice and littermate non-DREADD expressing controls. Decreasing the excitability of V2a neurons slowed the respiratory rate from 91.3 ± 4.3 to 53.5 ± 3.1 cycles/min (*n* = 6, *p* = 0.023; Fig. 2*D*) whereas no change was observed in non-DREADD controls (before CNO: 99.2 ± 5.1 cycles/min vs after CNO 94.0 ± 5.3 cycles/min, *n* = 5, *p* = 0.135; Fig. 2*F*). There is no significant difference in the respiratory rate between V2a-(G_i_)DREADD mice and controls before administration of CNO (*p* = 0.308).

To examine breath-to-breath consistency of the breathing frequency, we plotted the instantaneous frequency for at least 50 consecutive breaths before and after 10.0 mg/kg bw CNO treatment. V2a-(G_i_)DREADD mice injected with CNO showed a highly variable breathing frequency (Fig. 2*C*) that was not seen in CNO-treated non-DREADD controls (Fig. 2*E*). In addition, we generated Poincare maps by plotting the breathing period (Tn) versus the subsequent period (T_n+1_). When breathing is consistent, the points in a Poincare plot form a tight cluster (Del Negro et al., 2002; Crone et al., 2012), as seen in non-DREADD controls before and after administration of CNO (Fig. 2*J*). In contrast, decreasing the excitability of V2a neurons in neonatal V2a-(G_i_)DREADD mice resulted in disperse Poincare maps (Fig. 2*G*), indicating that breathing period fluctuates from breath to breath, similar to mice in which V2a neurons are ablated (Crone et al., 2012). To further quantify the regularity of breathing in V2a-(G_i_)DREADD mice, we compared the CV_f_ in each animal before and after CNO treatment. We also calculated the IS, which has previously been used to measure changes in respiratory rhythm regularity in medullary slices treated with pharmacological agents and whole animals following carotid body ablation (Viemari et al., 2011; Sheikhbahaei et al., 2017). The CV_f_ and IS were both significantly increased following CNO treatment in V2a-(G_i_)DREADD mice (CV_f_: 0.22 ± 0.03 vehicle vs 0.73 ± 0.15 CNO, *p* = 0.030, *n* = 6; IS: 19.22 ± 2.79% vehicle vs 68.81 ± 19.40% CNO, *p* = 0.031, *n* = 5; Fig. 2*H*,*I*). This is compared to no change observed in non-DREADD expressing control mice (CV_f_: 0.23 ± 0.03 vehicle vs 0.26 ± 0.02 CNO, *p* = 0.619, *n* = 6 IS: 16.67 ± 3.31% vehicle vs 21.65 ± 3.31% CNO, *p* = 0.363, *n* = 5; Fig. 2*K*,*L*). No fatalities occurred in either genotype following CNO treatment, despite the occurrence of apneas in V2a-(Gi)DREADD pups. Thus, V2a neuron activity is required to maintain both the frequency and regularity of breathing in neonatal mice.

### Decreasing V2a neuron excitability increases breathing frequency without altering regularity in adult mice

**Figure 3.**
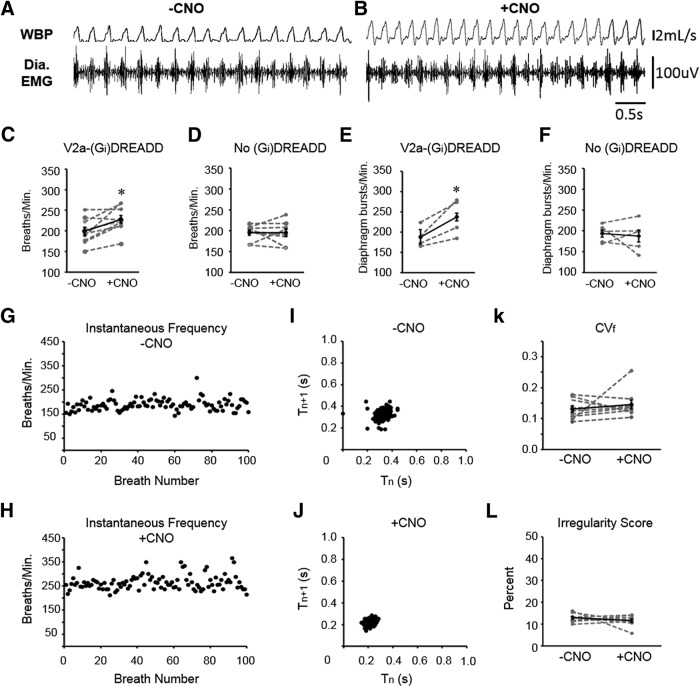
Decreasing the excitability of V2a neurons increases breathing frequency without causing irregular breathing in adult V2a-(G_i_)DREADD mice. ***A***, ***B***, Representative traces of WBP and diaphragm EMG following injections of vehicle (***A***) and subsequently 10.0 mg/kg bw CNO (***B***) into the same V2a-(G_i_)DREADD mouse on the same day. ***C***, ***D***, Instantaneous respiratory frequency was assessed in V2a-(G_i_)DREADD mice (**C**) and non-DREADD (Chx10^Cre/+^) control mice (***D***) by WBP after injection of vehicle (-CNO) and CNO. ***E***, ***F***, The instantaneous bursting frequency was calculated from the RMS^peak^ of the diaphragm EMG signal in V2a-(G_i_)DREADD mice (***E***) and non-DREADD control mice (***F***) after injection of vehicle (-CNO) and CNO. ***G***, ***H***, Instantaneous respiratory frequency is plotted for 100 consecutive breaths in a V2a-(G_i_)DREADD mouse injected with vehicle (***G***) or 10.0 mg/kg bw CNO (***H***) to illustrate the breath to breath consistency of respiratory frequency. ***I***, ***J***, Poincare maps generated by plotting the respiratory cycle period (T_n_) versus the subsequent respiratory cycle period (T_n+1_) from the same trials shown in ***G***, ***H*** show a consistent period following both vehicle (***I***) and CNO (***J***) treatments. ***K***, CNO treatment (10.0 mg/kg bw) does not alter the CV_f_ compared to vehicle (-CNO) treatment in V2a-(G_i_)DREADD mice (*p* = 0.492, *n* = 9). ***L***, The average breathing frequency IS of V2a-(G_i_)DREADD mice following vehicle and CNO (10.0 mg/kg bw) is not significantly different (*p* = 0.378, *n* = 9). Gray dashed lines show individual animals. Solid black line shows the mean ± SE; *p < 0.05, paired *t* test or Wilcoxon signed-rank test (Fig. 2*K*).

We next assessed whether acutely silencing V2a neurons in adult mice alters the frequency or regularity of breathing. Following intraperitoneal injection of either vehicle or CNO, V2a-(G_i_)DREADD mice (*n* = 9) were placed in a plethysmography chamber to measure ventilation. A subset of V2a-(Gi)DREADD mice (four of the nine) were instrumented with telemetry devices to record EMG from the diaphragm. A representative trace showing simultaneous recordings of WBP and diaphragm EMG following vehicle and CNO injections is shown in Figure 3*A*,*B*. Instantaneous breathing frequency (f) was measured from plethysmography recordings taken before and after CNO treatment (*n* = 9). Surprisingly, silencing V2a neurons in adult mice increased respiratory frequency 15.3% (Fig. 3*C*), whereas no change was observed in the control mice lacking the (G_i_)DREADD receptor (0.2 ± 4.5%; *p* = 1.00, *n* = 8; Fig. 3*D*). Consistent with WBP, the instantaneous bursting frequency of the diaphragm in V2a-(G_i_)DREADD mice was significantly increased after CNO treatment (188.0 ± 11.4 vehicle vs 237.553 ± 20.2 bursts/min CNO, *p* = 0.04, *n* = 4; Fig. 3*E*). No change was observed after CNO treatment in non-DREADD controls (193.7 ± 8.6 vehicle vs 187.7 ± 14.6 bursts/min CNO, *p* = 0.733, *n* = 5; Fig. 3*F*). There is no difference in bursting frequency between genotypes following administration of vehicle only (*p* = 0.735). Thus, adult mice show a small increase in breathing frequency when V2a neurons are silenced rather than a dramatic decrease in frequency observed in neonatal mice.

We next assessed whether silencing V2a neurons impacts the regularity of breathing in adult V2a-(G_i_)DREADD mice. To exclude potential effects caused by changes in ARM activity (see “Decreasing V2a neuron excitability increases ARM EMG activity”), we plotted the instantaneous frequency for 100 consecutive breaths during intervals in which ARMs are not active (interbout intervals). We observed a stable, consistent breathing frequency for each animal during vehicle and CNO treatment (Fig. 3*G*,*H*). Breathing regularity is evident from Poincare plots that form a tight cluster in vehicle and CNO-treated V2a-(G_i_)DREADD mice (Fig. 2*I*,*J*) as well as no effect of CNO treatment on the CV_f_ (0.146 ± 0.014 vehicle vs 0.130 ± 0.010 CNO, *p* = 0.492, *n* = 9; Fig. 3*K*) or IS (12.8 ± 0.6% vehicle vs 11.6 ± 0.8% CNO, *p* = 0.378, *n* = 9; Fig. 3*L*). These results demonstrate that, unlike in neonatal mice, V2a neuron activity is not critical for maintaining a regular respiratory rhythm in adult mice.

### Decreasing V2a neuron excitability increases ARM EMG activity

**Figure 4.**
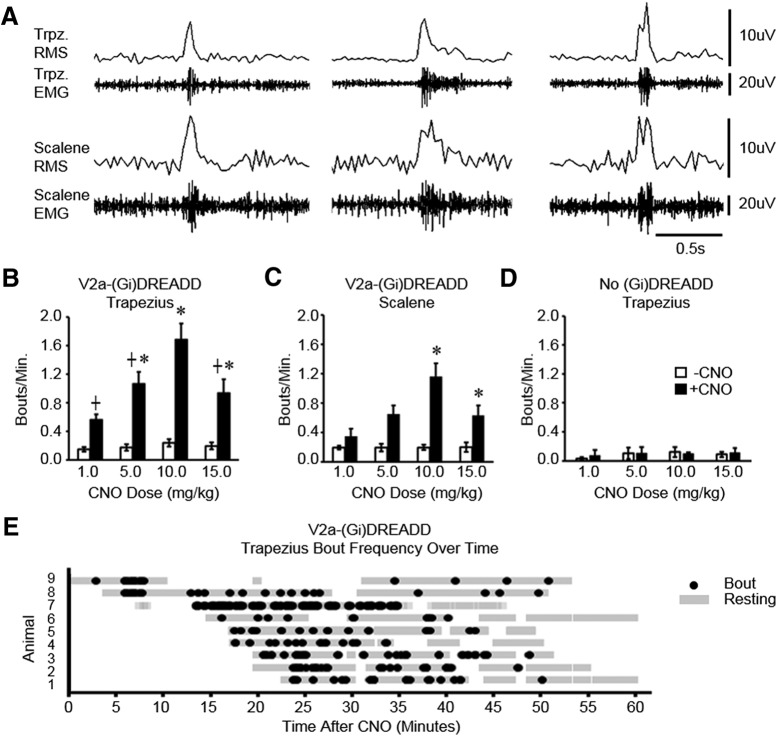
Decreasing the excitability of V2a neurons increases extradiaphragmatic respiratory muscle activity. ***A***, Representative traces showing three different examples of bouts of trapezius and scalene EMG activity (recorded simultaneously) following injection of 10.0 mg/kg bw CNO into a V2a-(G_i_)DREADD mouse. The RMS of the EMG signal is shown above the EMG trace for each muscle. The frequency of trapezius (***B***; *n* = 9) and scalene (***C***; *n* = 5) bouts was measured before CNO treatment (white bars) as well as following 1.0–15.0 mg/kg bw CNO (black bars) in V2a-(G_i_)DREADD mice. Experiments using different doses of CNO were performed on separate days within the same animals. ***D***, CNO treatment (1.0–15.0 mg/kg bw) does not increase trapezius bout frequency in non-DREADD control (Chx10^Cre/+^) mice lacking the V2a-(G_i_)DREADD receptor (*n* = 5). ***E***, The time point when each ARM bout occurred for the trapezius (*n* = 9) is plotted for each mouse (black dot) across the hour-long EMG recording following CNO treatment. Gray highlighted regions represent time periods each mouse spent in the resting state when ARM bout frequency could be analyzed; *, treatment group is significantly different from vehicle control (*p* < 0.05, one-way repeated measures ANOVA *p* < 0.05); ┼, group is different from 10.0 mg/kg bw CNO (*p* < 0.05, one-way repeated measures ANOVA). Gray dashed lines show individual animals. Solid black line shows the mean ± SE.

We next assessed the effect of decreasing V2a excitability on extradiaphragmatic respiratory muscle activity. Mice were instrumented with devices to non-invasively measure EMG from trapezius (*n* = 9) and either scalene (*n* = 5) or diaphragm (*n* = 4) muscles. V2a-(G_i_)DREADD mice were placed in a plethysmography chamber and muscle activity was measured after a vehicle injection and then after injecting CNO. The RMS of the EMG signal was used to identify bouts of increased muscle activity in mice at rest (Mantilla et al., 2011). There was no change in the percentage of time spent in a resting state before and after silencing V2a neurons (47.3 ± 5.2% vehicle vs 50.7 ± 5.8% CNO, *n* = 9, *p* = 0.670). Unexpectedly, we observed an increased incidence of bouts of trapezius and scalene activity after V2a neurons are inhibited (Fig. 4*A*). The frequency of trapezius and scalene bouts is dependent on the dose of CNO, with the highest frequency in both muscles observed at a dose of 10.0 mg/kg bw CNO (Fig. 4*B*,*C*). A 9.3-fold increase in trapezius and 7.5-fold increase in scalene activity was observed following CNO treatment at this dose. Trapezius and scalene muscles were usually co-activated (87 ± 4% of scalene bouts coincided with trapezius bouts, *n* = 5) following treatment with 10.0 mg/kg bw CNO, signifying that the bouts are a form of patterned activity. CNO does not increase trapezius or scalene activity in non-DREADD expressing control mice at any dose tested (Fig. 4*D*), demonstrating that the effects of CNO are the result of (G_i_)DREADD inhibition of V2a neurons. We observed a small but statistically significant difference in baseline trapezius ARM activity in V2a-(G_i_)DREADD mice not treated with CNO compared to non-DREADD controls at rest [0.203 ± 0.04 bouts/min (G_i_)DREADD (*n* = 9) vs 0.080 ± 0.021 bouts/min control (*n* = 5), *p* = 0.043]. However, a difference of 0.1 bouts/min is not biologically significant compared to the increase we see after CNO administration (1.46 bouts/min). Further, trapezius ARM activity in vehicle-treated V2a(G_i_)DREADD mice is comparable to what has previously been reported in wild-type mice (0.176 ± 0.038 bouts/min; Romer et al., 2017), indicating that this group of non-DREADD control mice had a particularly low level of baseline ARM activity. Finally, we plotted the occurrence of each trapezius bout over the hour-long recording period for each animal to characterize the timing of ARM activation, with time spent in the resting state indicated with gray bars (Fig. 4*E*). Results show that ARM bouts can occur as soon as 5 min following CNO treatment (accounting for the 2-min reacclimation to the chamber following CNO injection) and substantially declines within the last 15 min of the hour-long recording. In summary, inhibition of V2a neuron activity causes an increase in the incidence of trapezius and scalene muscle activity in mice at rest.

### Diaphragm EMG peak amplitude is not altered when V2a excitability is decreased

**Figure 5.**
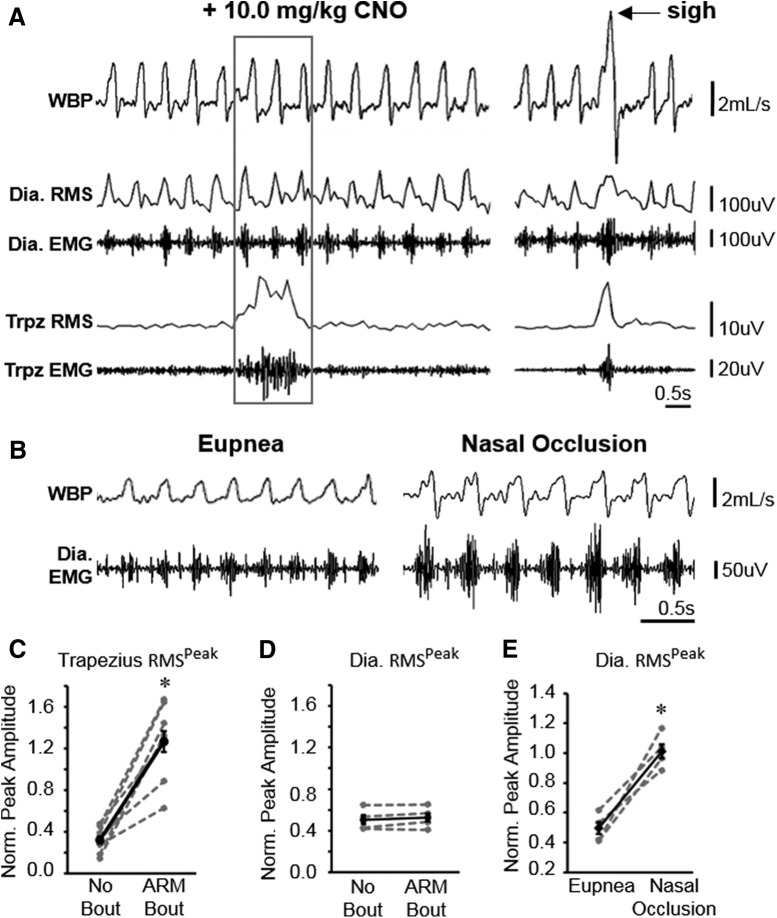
Diaphragm EMG peak amplitude does not change during ARM bouts. ***A***, Representative traces showing WBP as well as diaphragm and trapezius EMG signals recorded simultaneously from a V2a-(G_i_)DREADD mouse. The RMS of the EMG signal is shown above the EMG trace for each muscle. Left, The gray box indicates a bout of trapezius activity in a CNO-treated mouse. Right, WBP, EMG, and RMS signals from the same mouse during a sigh (near maximal ventilation, arrow). ***B***, Representative trace showing WBP and diaphragm EMG during eupnea and during nasal occlusion. ***C***, ***D***, Diaphragm activity is not increased during bouts of increased trapezius activity. ***C***, Trapezius RMS^peak^ values were normalized to trapezius RMS^peak^ values during sighs to calculate the average normalized peak amplitude of trapezius activity for each animal (*n* = 9 animals) during bouts of trapezius activity (ARM bout) and during interbout intervals (no bout). ***D***, Diaphragm RMS^peak^ during bouts of trapezius activity and during interbout intervals, normalized to diaphragm RMS^peak^ during sighs for each animal (*n* = 4). ***E***, Diaphragm RMS^peak^ is increased during nasal occlusion (*n* = 4, *p < 0.05, paired *t* test). Gray dashed lines represent individual animals. Solid black lines show the mean ± SE for all animals.

To address the possibility that increased ARM activity resulting from silencing V2a neurons is a compensatory response to impaired diaphragm function, we analyzed the diaphragm EMG signal in V2a-(G_i_)DREADD mice and controls implanted with leads in the diaphragm and trapezius muscles (Fig. 5*A*). The peak amplitude of the RMS of the EMG signal (RMS^peak^) was used to measure the amplitude of each burst of diaphragm activity (each breath) as well as trapezius activity during bouts and interbout intervals. The RMS^peak^ amplitude was normalized to the amplitude during near maximal ventilatory behavior (sighs) for each muscle to reduce intra-animal variability (Mantilla et al., 2011). The trapezius RMS^peak^ is not significantly different between animals implanted in the trapezius and diaphragm (*n* = 4) compared to trapezius and scalene (*n* = 5; *t* test *p* = 0.938), so these two groups were pooled for analysis of trapezius RMS^peak^. We observe a 4.2-fold increase in trapezius RMS^peak^ during a bout compared to interbout intervals (Fig. 5*C*). In contrast, we observe no significant change in diaphragm RMS^peak^ during bouts of trapezius activity compared to interbout intervals (Fig. 5*D*) and no change in diaphragm RMS^peak^ during interbout intervals following CNO injection compared to vehicle only (0.51 ± 0.05 vehicle vs 0.52 ± 0.05 CNO, *n* = 4, paired *t* test *p* = 0.889). However, we verified that our devices are capable of measuring changes in diaphragm RMS^peak^ during different behaviors by performing nasal occlusion for 4 min on the same V2a-(G_i_)DREADD mice (Fig. 5*B*). Nasal occlusion is known to increase ventilatory drive and increase diaphragm RMS^peak^ (Mantilla et al., 2011). We measured a 2.1-fold increase in diaphragm RMS^peak^ following nasal occlusion compared to eupnea (Fig. 5*E*), consistent with previously published literature. Thus, despite our ability to detect increases in the peak amplitude of diaphragm muscle activity during sighs and nasal occlusion, we did not observe a change in diaphragm peak amplitude in V2a-(G_i_)DREADD mice following CNO treatment.

### Decreasing V2a neuron excitability increases ventilation

**Figure 6.**
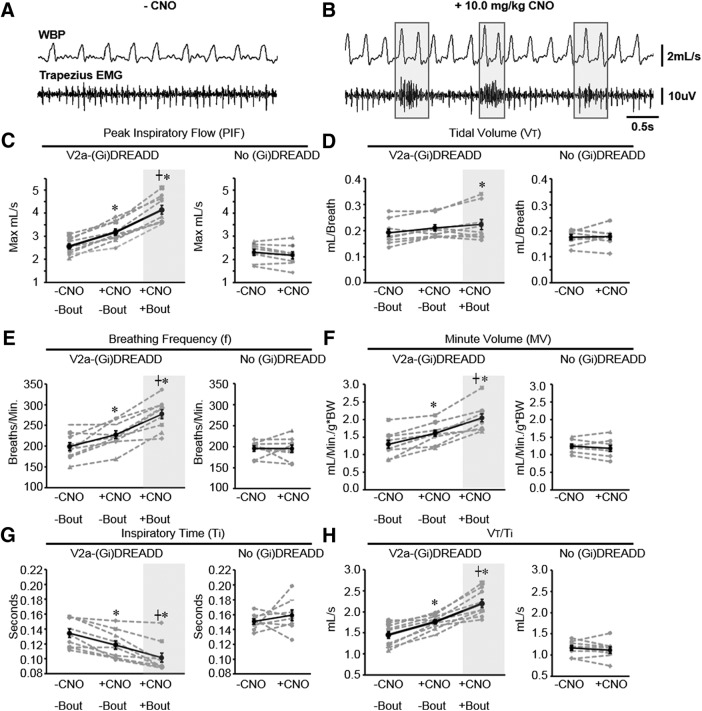
Ventilation is increased during bouts of ARM activity. ***A***, Representative trace showing WBP and trapezius EMG following injection of vehicle. ***B***, Representative trace showing WBP and trapezius EMG after injection of 10.0 mg/kg bw CNO. Gray boxes outline trapezius bouts and corresponding changes in WBP. PIF (***C***), V_T_ (***D***), breathing frequency (BPM; ***E***), MV (***F***), T_i_ (***G***), and V_T_/T_i_ (***H***) were measured within the same V2a-(G_i_)DREADD mice (*n* = 9) and non-DREADD control mice (*n* = 8) during interbout intervals in the absence of CNO, during interbout intervals with 10.0 mg/kg bw CNO, and during trapezius bouts with 10.0 mg/kg bw CNO. The low frequency of ARM activity in non-DREADD controls precluded measurement of WBP during ARM bouts; *, group is different from -CNO/-bout (vehicle control); ┼, +CNO/+bout is different from +CNO/-bout (*p* < 0.05, one-way repeated measures ANOVA with all comparisons Holm–Sidak *post hoc* test for V2a-(G_i_)DREADD mice or paired *t* test for non-DREADD controls). Gray dashed lines show individual animals. Solid black line shows the mean ± SE.

To assess the impact on ventilation of decreasing V2a neuron excitability, we analyzed WBP to compare ventilation following CNO injection to vehicle control injections. We first focused our analysis on interbout intervals to exclude potential effects on ventilation caused by increased ARM recruitment. We observed a 24.6% increase in PIF, 28.6% increase in MV, and 15.3% increase in breathing frequency (**f**) in V2a-(G_i_)DREADD mice after silencing V2a neurons, but no change was observed in V_T_ (Fig. 6*A–F*). In addition, there was no significant difference in sigh frequency following CNO injection compared to vehicle (0.54 ± 0.14 vehicle vs 0.54 ± 0.05 CNO; *p* = 0.812). We assessed ventilatory drive by measuring V_T_/T_i_ (Morgan et al., 2014) and found that it is increased following CNO treatment, primarily driven by a decrease in T_i_ (Fig. 6*G*,*H*). Importantly, the effects of CNO on ventilation are caused by altering G_i_ protein signaling in V2a neurons because we observed no CNO-dependent changes in PIF, V_T_, MV, or f in non-DREADD control mice (Fig. 6*C–F*). We also investigated any potential effects of (G_i_)DREADD expression alone on breathing by comparing ventilation parameters in V2a-(G_i_)DREADD and non-DREADD controls following vehicle injection. We did not find a statistically significant difference in PIF (V2a-(G_i_)DREADD: 2.562 ± 0.120 vs non-DREADD 2.306 ± 0.139; *p* = 0.182), V_T_ (0.192 ± 0.014 vs 0.176 ± 0.010; *p* = 0.716), MV (1.24 ± 0.06 vs 1.29 ± 0.10; *p* = 0.728), f (199.2 ± 9.4 vs 195.6 ± 6.3; *p* = 0.066), or Ti (0.134 ± 0.006 vs 0.151 ± 0.004; *p* = 0.136). Our results are the first to demonstrate that inhibiting V2a neurons in adult animals impacts ventilation, even during the intervals between bouts of ARM activity.

We further investigated the impact of increased ARM activity on breathing by comparing ventilation during bouts of trapezius activity to ventilation during interbout intervals in V2a-(G_i_)DREADD mice implanted with leads in the diaphragm and trapezius (*n* = 4). Significant increases are observed in PIF (30.6%), MV (28.0%), and f (22.2%) during ARM bouts compared to interbout intervals in V2a-(G_i_)DREADD mice treated with CNO (Fig. 6*C*,*E*,*F*). There is a modest (15.6 ± 2.9%) increase in V_T_ during ARM bouts that is statistically significant compared to V_T_ during interbout intervals in the absence of CNO (Fig. 6*D*). V_T_/T_i_ is increased during ARM bouts, primarily due to a significant decrease in T_i_ (Fig. 6*G*,*H*). The decrease in T_i_ and increase in f during bouts is consistent with a decrease in diaphragm EMG burst duration (64 ± 3 ms bout, 97 ± 3 ms no bout; *p* = 0.003) and increase in f (298 ± 10 bursts/min bout, 238 ± 18 no bout; *p* = 0.012) following CNO injections observed in V2a-(G_i_)DREADD mice. We did not attempt to quantify ventilatory parameters during trapezius activity in non-DREADD expressing mice or V2a-(Gi)DREADD mice during vehicle injections due to the rarity of ARM bouts in control mice. These data show that the ARM activity resulting from inhibition of V2a neurons is associated with a further increase in ventilation over that observed during interbout intervals.

To determine whether ARM activity might be triggered by a change in ventilation, we compared MV during the four breaths immediately preceding each bout of ARM activity to the average MV during interbout intervals and found no significant difference (1.52 ± 0.06 vs 1.61 ± 0.09 ml/min per g/bw; *p* = 0.392). We also detected no significant differences in PIF (3.22 ± 0.11 vs 3.18 ± 0.14 max ml/s; *p* = 0.823), VT (0.19 ± 0.01 vs 0.21 ± 0.01 ml/breath; *p* = 0.282), or f (227.1 ± 9.8 vs 227.7 ± 9.7 breaths/min; *p* = 0.922) during breaths immediately preceding a bout compared to interbout intervals. However, we did observe a small but significant decrease in T_i_ immediately preceding a bout (0.11 ± 0.01 vs 0.19 ± 0.01 s; *p* = 0.030). Thus, the majority of ventilation parameters are not significantly altered immediately before the onset of ARM bouts.

### Increasing and decreasing the excitability of V2a neurons differentially affects motor functions

**Figure 7.**
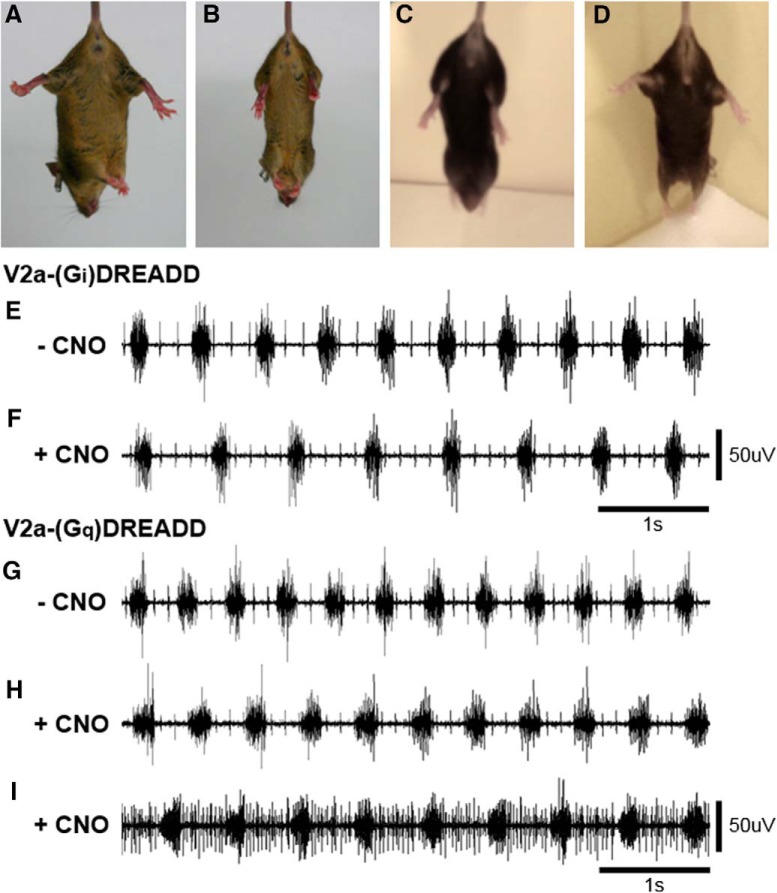
Increasing and decreasing the excitability of V2a neurons differentially affects motor functions. ***A–D***, Mice were suspended by the tail to assess hindlimb extension. ***A***, Wild-type mice extend their hindlimbs. ***B***, Chx10::DTA mice (in which V2a neuron were ablated during development) do not splay their hindlimbs. ***C***, V2a-(G_i_)DREADD mice treated with 10 mg/kg bw CNO do not splay their hindlimbs. ***D***, V2a-(G_q_)DREADD mice treated with 1.0 mg/kg bw CNO splay their hindlimbs. ***E–I***, Diaphragm EMG was recorded under isoflurane anesthesia in V2a-(G_i_)DREADD and V2a-(G_q_)DREADD mice. Normal diaphragm bursting activity is observed in V2a-(G_i_)DREADD mice before (***E***) and after (***F***) silencing V2a neurons with 10 mg/kg bw CNO, as well as in V2a-(G_q_)DREADD mice before CNO treatment (***G***). V2a-(G_q_)DREADD mice treated with 1.0 mg/kg bw CNO alternate between a normal diaphragm bursting pattern (***H***) and a pattern that also includes diaphragm activity during the expiratory phase (***I***).

It has previously been shown that increasing the excitability of V2a neurons with CNO in V2a-(G_q_)DREADD mice activates ARMs (Romer et al., 2017). Since increasing and decreasing the excitability of V2a neurons both increase the incidence of ARM bouts, we examined the effects of altering the excitability of V2a neurons on other motor activities. Wild-type mice can fully splay their hindlimbs when lifted by the tail (Fig. 7*A*), whereas mice lacking V2a neurons do not extend their hindlimbs (*n* = 3; Fig. 7*B*). Before CNO treatment, V2a-(G_i_)DREADD mice fully extend their hindlimbs when suspended by the tail, like wild-type mice. However, following CNO treatment (10 mg/kg bw), V2a-(G_i_)DREADD mice (*n* = 3) lose this hindlimb extension reflex and their limbs hang in a relaxed manner (Fig. 7*C*). V2a-(G_q_)DREADD mice (*n* = 3) show normal hindlimb extension before and after treatment with CNO (1.0 mg/kg bw; Fig. 7*D*). The selected doses of CNO were those that elicited the greatest increase in ARM activity for each mouse line. Thus, increasing and decreasing the excitability of V2a neurons has distinct effects on a hindlimb extension reflex.

Next, we examined the effects of increasing and decreasing the excitability of V2a neurons on diaphragm activity by performing terminal diaphragm EMG recordings under isoflurane anesthesia. Similar to what is observed in conscious V2a-(G_i_)DREADD mice instrumented with telemetry transmitters, silencing V2a neurons does not alter the diaphragm EMG peak amplitude (0.61 ± 0.07 vehicle vs 0.52 ± 0.03 CNO, *n* = 3, paired *t* test, *p* = 0.376) or regularity of diaphragm bursting in anesthetized mice (CV_f_: 0.04 ± 0.004 vehicle vs 0.04 ± 0.003 CNO, *n* = 3, paired *t* test, *p* = 0.903; IS: 3.94 ± 0.27 vehicle vs 3.97 ± 0.27 CNO, *n* = 3, paired *t* test, *p* = 0.935; Fig. 7*E*,*F*). We then used the same protocol to assess diaphragm EMG activity after increasing the excitability of V2a neurons in V2a-(G_q_)DREADD mice. CNO does not alter the normalized diaphragm EMG peak amplitude in anesthetized mice (0.46 ± 0.04 pre CNO vs 0.44 ± 0.03 post CNO, *n* = 5, paired *t* test, *p* = 0.984; Fig. 7*G–I*). However, we observed additional activity during the expiratory phase of the respiratory cycle in 5 out of 5 V2a-(G_q_)DREADD mice (Fig. 7*I*). Expiratory diaphragm activity is observed intermittently during 44 ± 8% of the total recording time following CNO treatment with an average duration of 308 ± 112 s. In contrast, activity during the expiratory phase is not observed in V2a-(G_i_)DREADD mice (*n* = 3; Fig. 7*F*). These results demonstrate that although increasing and decreasing the excitability of V2a neurons has similar effects on ARM activity, the two manipulations have distinct effects on diaphragm activity during the expiratory phase of respiration.

## Discussion

In this study, we tested the role of brainstem and spinal cord V2a neurons in respiratory rhythm generation and pattern formation by acutely silencing V2a neurons in conscious neonatal or adult mice using an inhibitory DREADD while measuring respiratory muscle activity and ventilation. We show that V2a neural activity is required to maintain the frequency and regularity of breathing in neonatal mice. However, V2a neuron activity is not required to maintain respiratory rhythm regularity in adult mice. In addition, we show that decreasing the excitability of V2a neurons results in activation of extradiaphragmatic respiratory muscles and increased ventilation, with little effect on diaphragm activity. Thus, during the neonatal period V2a neuron function is critical for respiratory rhythm generation, whereas in adults V2a neurons play an important role in patterning the activity of extradiaphragmatic respiratory muscles.

Our study also suggests that inhibiting V2a neuron activity could be a safe, effective method to increase ARM activity and ventilation without impairing diaphragm function in adult patients with neuromuscular disease or spinal cord injury.

This study is the first to demonstrate that acutely silencing V2a neurons in neonatal mice causes slow and irregular breathing. A limitation of a prior study in which V2a neurons were ablated during development (Crone et al., 2012) is that it was not possible to distinguish between the acute functions of V2a neurons in neonatal mice versus potential developmental defects that might have resulted from the loss of V2a neurons in the embryo. Importantly, CNO does not alter the frequency or regularity of breathing in non-DREADD expressing mice, demonstrating that our results are not due to off-target effects of CNO on other cell types. Further, we observe no difference in breathing frequency or pattern in V2a-(G_i_)DREADD mice and non-DREADD control mice before CNO treatment, indicating that DREADD expression alone does not appear to impair the development or function of V2a neurons. Within the medulla, V2a neurons are located in the medial reticular formation and are distinct from several other classes of neurons known to influence respiration, including the Dbx1^+^ V0 neurons of the ventral respiratory group, Phox2b^+^ and Atoh1^+^ neurons of retrotrapezoid nucleus/parafacial respiratory group, Lmx1b^+^ and Pet1^+^ catecholaminergic and serotonergic neurons, and Pax2^+^, Lbx1^+^, and FoxP2^+^ neurons of the brainstem (Gray, 2013; Dick et al., 2018). Our study is consistent with prior studies indicating that V2a neurons in the medial reticular formation of the brainstem provide excitatory drive to neurons in the pre-Bötzinger complex (likely V0 neurons) required for normal respiratory rhythm generation in neonatal mice (Crone et al., 2012).

By performing acute silencing experiments in adult mice, we show that V2a neuron activity is not required in mature animals for regular breathing rhythm. In fact, instead of slowing respiration, we observe a small increase in frequency when V2a neurons are silenced in the adult. Prior studies in which V2a neurons were ablated could not distinguish whether the recovery of a normal breathing rhythm after the first week of birth was because V2a neurons are no longer needed at older ages or the result of compensatory changes caused by ablation. Our results demonstrate that the role of V2a neurons in modulating respiratory rhythm changes during normal postnatal development. It is unlikely that the lack of slow and irregular breathing after silencing V2a neurons in adult mice is due to downregulation of the (G_i_)DREADD receptor in V2a neurons during maturation since there is no difference in the percentage of V2a neurons that express the (G_i_)DREADD receptor between neonatal and adult mice in both the brainstem and spinal cord. It is also unlikely that this difference is the result of dramatic changes in connectivity between V2a neurons and brainstem respiratory centers since increasing V2a neuron excitability can increase respiratory frequency in adult mice (Romer et al., 2017). Moreover, V2a neurons appear to project throughout the ventral respiratory column in adult mice (Romer et al., 2017; our unpublished observations). Many changes occur in the neural control of respiration during the perinatal period, including changes in chemosensation (Guyenet and Bayliss, 2015), neuromodulator signaling (Gray et al., 1999; Hilaire and Duron, 1999; Hilaire et al., 2004; Doi and Ramirez, 2008; Wong-Riley and Liu, 2008), inhibitory neurotransmission (Hübner et al., 2001; Okabe et al., 2015; Dubois et al., 2018), afferent regulation (Hilaire and Duron, 1999; Dutschmann et al., 2004), pontine-medullary interactions (Oku et al., 2007; Dutschmann and Dick, 2012; Del Negro et al., 2018), as well as morphology and properties of respiratory network neurons (Denavit-Saubié et al., 1994; Di Pasquale et al., 1996; Richter and Smith, 2014). Thus, the loss of excitatory drive provided by V2a neurons may be largely mitigated by other sources of drive in the adult that are not present or sufficient to restore breathing rhythm in newborn mice. Alternatively, drive from V2a neurons may only be required under specific conditions or behaviors in adult mice that were not tested in the present study (e.g., running).

Although we cannot rule out the possibility that the small population of V2a neurons that apparently lack DREADD expression are sufficient to maintain the frequency and regularity of breathing in adult mice, this seems unlikely given our results that this population is insufficient to maintain normal breathing in neonatal mice. Despite the fact that we are unable to detect DREADD expression in a significant fraction of medullary V2a neurons (35% of Chx10^+^ cells), we observe a strikingly similar severity of phenotype in neonatal V2a-(G_i_)DREADD mice treated with CNO as observed in Chx10::DTA mice in which >98% of V2a neurons were ablated during development (Crone et al., 2012). We do not know whether this is because silencing a majority of V2a neurons is sufficient to impair breathing or whether we are underestimating the fraction of V2a neurons expressing (G_i_)DREADD due to limitations in the sensitivity of our HA antibody. Further, we observe specific motor deficits (including failure of hindlimbs to splay when mice are suspended by the tail and increased ankle extension when walking) in adult V2a-(Gi)DREADD mice treated with CNO that are consistent with loss of V2a neuron function because they are also observed in mice in which V2a neurons are ablated (our unpublished observations).

Our study has also revealed an unexpected role for V2a neurons in constraining extradiaphragmatic respiratory muscle activity at rest. We observed coordinated activation of scalene and trapezius muscles at rest when V2a neurons were silenced. Interestingly, these results mimic the effects of activating V2a neurons at rest in healthy V2a-(G_q_)DREADD mice with the excitatory (G_q_)DREADD receptor (Romer et al., 2017). It is unlikely that one DREADD strategy (activating or silencing) is failing because we observe different effects of CNO on the diaphragm in V2a-(G_i_)DREADD mice versus V2a-(G_q_)DREADD mice. Specifically, activating V2a neurons causes activity during the expiratory phase of the respiratory cycle, whereas silencing V2a neurons does not. We also observe that V2a-(G_i_)DREADD mice, but not V2a-(G_q_)DREADD mice, fail to splay their legs when suspended by the tail after CNO treatment, a phenotype observed after V2a neurons have been ablated. Together, these results demonstrate that although silencing and activating V2a neurons both increase ARM activity in healthy mice, each manipulation has distinct effects on diaphragm activity and hindlimb reflexes.

Alternatively, it is possible that decreasing V2a neuron excitability impairs diaphragm function and thereby causes a compensatory increase in ARM activity. However, this mechanism is not consistent with our results as diaphragm RMS^peak^ is not altered during ARM bout activity or interbout intervals in V2a-(G_i_)DREADD mice treated with CNO. Thus, diaphragm function at rest does not appear to be impaired by silencing V2a neurons, although we cannot rule out a role for V2a neurons in modulating diaphragm activity during exercise, cough, or other behaviors. We do not think that this result is due to our inability to detect changes in diaphragm activity as we clearly see changes in diaphragm RMS^peak^ during sighs and following nasal occlusion, behaviors that require higher force generation by the diaphragm (Mantilla et al., 2011). We observed no changes in the frequency of sighs or arousals from sleep, as would be expected if animals experienced hypoxia or hypercapnia, and no changes in ventilation immediately preceding ARM activity. Thus, although we cannot rule out subtle changes in diaphragm function that are not detectable by our methods, it is unlikely that increased ARM activity is secondary to diaphragm impairment caused by silencing V2a neurons.

Another potential mechanism leading to ARM recruitment could be an increase in respiratory drive caused by silencing V2a neurons. We do, in fact, observe changes in ventilation even during periods in which ARMs are not active that are consistent with changes in the activity of brainstem respiratory networks, namely a higher frequency of breathing, shorter T_i_ and increased V_T_/T_i_ (an indicator of neural ventilatory drive). A higher PIF appears to compensate for the shorter T_i_ to maintain V_T_. We see an even greater increase in f, PIF, and V_T_/T_i_ during ARM activity compared to interbout intervals, as well as an increase in V_T_ (as expected when ARMs are recruited). However, it is not clear from these experiments if the ventilatory changes are due to direct actions of V2a neurons on brainstem respiratory centers or the result of chemosensory or sensory feedback. Despite the changes in respiratory timing, the phrenic motor neurons appear to receive similar levels of overall drive because we observe no detectable change in peak diaphragm activity when V2a neurons are silenced. Moreover, the peak diaphragm activity does not change even when ARMs become active, suggesting that the increased ARM activity is driven by circuits independent of (or with minimal impact on) diaphragm activity. This concept is consistent with studies in human patients showing that neural drive is not uniform to all inspiratory muscles, and may include inspiratory, expiratory and tonic components differentially distributed across motor neuron pools (Butler, 2007; Butler et al., 2014).

A previous study demonstrated that increasing the excitability of V2a neurons is able to activate ARMs (Romer et al., 2017). To explain our findings that either increasing or decreasing V2a excitability can increase ARM activity, we hypothesize that a distinct subset of V2a neurons promotes ARM activity (i.e., during exercise) whereas another distinct subset inhibits ARM activity at rest (i.e., to prevent activation when they are not needed). An inhibitory pathway for ARMs may serve to reduce unnecessary (and energetically expensive) respiratory muscle activity during behaviors that require low force generation when diaphragm activity is sufficient. The reticulospinal system provides another example where different subsets of V2a neurons appear to produce distinct effects on motor activity. Activating V2a neurons in the rostral medulla can halt ongoing locomotion, indicating that these neurons inhibit locomotor circuits (Bouvier et al., 2015). On the other hand, ablating V2a neurons prevents initiation of fictive locomotion following stimulation of the caudal medulla in a neonatal brainstem-spinal cord preparation, indicating that caudal medullary or spinal V2a are important for activating locomotor circuits (Crone et al., 2008). Further, even within spinal locomotor circuits, different molecularly defined subsets of V2a neurons appear to have different roles in controlling locomotion. For example, Shox2^+^/Chx10^+^ V2a neurons provide excitatory drive to ipsilateral limb motor neurons important for maintaining consistent motor output whereas Shox2^-^/Chx10^+^ V2a neurons have connections to inhibitory commissural neurons that maintain alternation of left and right sides at high locomotor speeds (Dougherty et al., 2013; Rybak et al., 2015). An alternative hypothesis is that silencing V2a neurons induces a compensatory change in downstream neurons (i.e., respiratory motor neurons) which renders them more excitable to other inputs, leading to ARM activation. Distinguishing between these two possibilities would require identifying specific V2a subsets that only activate ARMs when silenced but not activated (or vice versa) or demonstrating that either silencing or inhibiting the same pre-motor V2a neurons could activate ARMs.

An important limitation of this study is that our mouse model targets V2a neurons in both the brainstem and spinal cord, two populations that likely play different roles in the control of breathing. Crone et al. (2012) suggested that V2a neurons in the medulla provide excitatory drive necessary for respiratory rhythm generation, since pontine and spinal cord neurons are not present in the transverse medullary slice preparation used in some experiments. However, they did not rule out additional roles for pontine or spinal V2a neurons, and neither do the experiments reported here. Additional studies using transverse medullary slices or pontomedullary preparations from neonatal V2a-(G_i_)DREADD mice could further test the importance of brainstem V2a neurons for respiratory rhythm generation as well as investigate the mechanism(s) by which excitatory drive from these neurons promotes frequent, regular inspiratory burst activity. Preparations from older animals could be used to further investigate whether V2a neurons have a less significant impact on inspiratory rhythm generating networks at older ages or whether other neurons/structures compensate for the loss of excitatory drive from V2a neurons. We hypothesize that spinal V2a neurons may be responsible for patterning respiratory motor output, including control of ARM activity. However, we cannot rule out a role for brainstem neurons, such as reticulospinal V2a neurons, in either promoting or inhibiting ARM activity. Future experiments using viruses to target excitatory or inhibitory DREADDs to only cervical V2a neurons or only specific brainstem regions could map the location of V2a neurons controlling ARM activity. Likewise, intersectional genetic approaches that require Cre and Flp recombinases to drive DREADD expression (Ray et al., 2011) could be used to target only subsets of spinal or brainstem V2a neurons to test their roles in controlling respiratory rhythm and pattern. A better grasp of what different subtypes of V2a neurons do within respiratory circuits will advance our understanding of how spinal and brainstem circuits regulate breathing.

Our findings suggest that approaches to either activate or inhibit V2a neurons could increase ARM activity and enhance respiration in patients with neuromuscular disease or spinal cord injury. The best therapeutic approach (activation or inhibition) may depend on which subset of V2a neurons is targeted, as well as assessing the potential for undesirable side effects. Future experiments targeting subpopulations of V2a neurons in different regions of the brainstem and spinal cord and/or with different transcriptional profiles may help resolve the distinct roles of different V2a subtypes and facilitate identifying therapeutic targets to improve breathing.

**Table 1.** Summary of statistical analyses

Data	Structure	Statistical test	Power/95% confidence interval
Figure 2*D*	Parametric	Paired *t* test	C.I. 20.202 to 58.131
Figure 2*F*	Parametric	Paired *t* test	C.I. –2.524 to 12.924
Figure 2*H*	Parametric	Paired *t* test	C.I. –0.947 to –0.0760
Figure 2*I*	Non-parametric	Wilcoxon signed-rank test	C.I. 13.301–79.387
Figure 2*K*	Parametric	Paired *t* test	C.I. –0.140 to 0.0948
Figure 2*L*	Parametric	Paired *t* test	C.I. –18.462 to 8.502
Figure 3*C*	Parametric	Paired *t* test	C.I. –48.415 to –8.548
Figure 3*D*	Parametric	Paired *t* test	C.I. –21.236 to 21.966
Figure 3*E*	Parametric	Paired *t* test	C.I. –96.817 to –2.286
Figure 3*F*	Parametric	Paired *t* test	C.I. –39.657 to 51.697
Figure 3*K*	Non-parametric	Wilcoxon signed-rank test	C.I. 0.102–0.153
Figure 3*L*	Parametric	Paired *t* test	C.I. –1.856 to 4.376
Figure 4*B*	Parametric	One-way RM ANOVA	Power: 1.000
Figure 4*C*	Parametric	One-way RM ANOVA	Power: 0.991
Figure 4*D*	Parametric	One-way RM ANOVA	Power: 0.500
Figure 5*C*	Parametric	Paired *t* test	C.I. –1.199 to –0.688
Figure 5*D*	Parametric	Paired *t* test	C.I. –0.0680 to 0.0273
Figure 5*E*	Parametric	Paired *t* test	C.I. –0.795 to –0.240
Figure 6*C*, DREADD	Parametric	One-way RM ANOVA	Power: 1.000
Figure 6*C*, no DREADD	Parametric	Paired *t* test	C.I. –0.0229 to 0.280
Figure 6*D*, DREADD	Parametric	One-way RM ANOVA	Power: 0.804
Figure 6*D*, no DREADD	Parametric	Paired *t* test	C.I. –0.0232 to 0.0198
Figure 6*E*, DREADD	Parametric	One-way RM ANOVA	Power: 1.000
Figure 6*E*, no DREADD	Parametric	Paired *t* test	C.I. –21.236 to 21.966
Figure 6*F*, DREADD	Parametric	One-way RM ANOVA	Power: 1.000
Figure 6*F*, no DREADD	Parametric	Paired *t* test	C.I. –0.0547 to 0.175
Figure 6*G*, DREADD	Parametric	One-way RM ANOVA	Power: 1.000
Figure 6*G*, no DREADD	Parametric	Paired *t* test	C.I. –0.0299 to 0.0134
Figure 6*H*, DREADD	Parametric	One-way RM ANOVA	Power: 1.000
Figure 6*H*, no DREADD	Parametric	Paired *t* test	C.I. –0.0645 to 0.163

## Synthesis

Reviewing Editor: Muriel Thoby-Brisson, CNRS UMR 5287 Université Bordeaux Decisions are customarily a result of the Reviewing Editor and the peer reviewers coming together and discussing their recommendations until a consensus is reached. When revisions are invited, a fact-based synthesis statement explaining their decision and outlining what is needed to prepare a revision will be listed below. The following reviewer(s) agreed to reveal their identity: Michael Lane, Julien Bouvier.

This manuscript is a very well written report of respiratory function in neonatal and adult when V2a neuron activity is silenced. The impact of V2a neurons on respiration deserves examination since these neurons might modulate Accessory Respiratory Muscles (ARM), with potential benefits for enhancing respiration in ALS models. The transgenic animals used allow for the incorporation of the HM4di DREADD receptor into V2a (Chx10-expressing) neurons. The experiments are well described and reveal quite un-expected and interesting results that are valuable and extremely important to the field of respiratory neurobiology.

However some key issues must be addressed to make effective and accurate interpretations of the results presented. For instance:

-1) The authors describe in detail the anatomical studies they performed, and the quantitative analysis made on the collected tissues. However, without any figures of this anatomy, it is very hard to put their results into context. Show the HA-tagged and the Chx10 labeled cells in the neonatal and adult tissue collected from both medulla and spinal cord. It will be important to see the cells described and quantified, and possibly their brainstem and spinal distributions if the tissue retained allows it. This will also better highlight differences between the 2 anatomical locations in the two developmental stages studied.

Could the authors please also clarify why only spinal cord sections from C4 were used for quantification (only even then only every 8th section), when spinal cord from C1 to C6 was sectioned. Was any software used to assist with analysis?

-2) The authors clearly document functional effects of silencing those cells expressing the DREADD receptor, but an important point to note is that neurons in both the medulla and spinal cord are affected by CNO delivery, and these are likely two quite different populations of neurons. This needs to be considered when then drawing conclusions about the contribution these cells make respiratory activity. The authors need to either i) address this with targeted silencing of one population over the other, or ii) carefully discuss their data, limitations and interpretations in light of this fact.

-3) In the adult, the increase of respiratory frequency and mobilization of accessory respiratory muscles bouts following V2a silencing is strikingly reminiscent of another study: if I understood right, Romer et al. (2017) reported very similar findings by activating the same neurons (e.g. compare current fig 4 with Fig 4 or Romer et al). How can an opposite interference of the same neurons' activity yield similar effects? The absence of mirror effects opens the possibility that one strategy (silencing or activating) is failing, which authors need to rule out. Also, the paper currently does not help to understand whether a possible strategy for enhancing respiratory function in ALS would be to favor, or on the contrary to inhibit, V2a neuronal activity.

a. Isn't it possible that over-activation may lead to inability to maintain spiking, or to change of the firing pattern, altogether leading to a functional inhibition? Authors should do the activation experiments themselves to verify the findings of the Romer paper in their own conditions, with appropriate CNO doses.

b. Alternatively, the timing might be important; however here there are no time-points following CNO administration and the timing used is not clearly stated. It seems that recordings are performed immediately after CNO administration, for 1h, is that right? However, several groups have reported peak effects at later time-points (between 1h and 2h). Can authors be more specific regarding time-points and check or report the effects over a longer period of time?

c. Finally, it could be expected that V2a inhibited mice have a dis-regulated baseline locomotor activity (Bretzner 2013; Bouvier 2015), with a possible indirect impact on ARM. Have authors controlled for this?

In any case, authors should also include a deep discussion paragraph on these discrepancies.

-4) Authors report that V2a inhibition increases breathing and diaphragm frequency by 15% (2C; 2E) but plots in 2G and 2H show a similar breathing frequency between the two groups throughout the recording. These plots of instantaneous frequency are not convincing (compare with the newborn situation, which is). In fact the value (~250 / min) is above average of the group data in 2C-F and seem to concern an animal that does not increase. Can authors verify their plots and/or bar-graphs and maybe select a trace that is a representative of the group data?

-5) Introduction, last paragraph, page 5: “we uncovered an unexpected role for V2a neurons in suppressing extradiaphragmatic activity at rest (...) circuit that actively prevents activation of ARMs”. This is not shown in the work. This would imply that activating V2a during ARM bouts suppresses them; this is not done here. Please remove/rephrase.

Minor comments :

- Please correct spelling of “pre-Boetzinger” to “pre-Bötzinger”

- The authors state that tissues were transcardially perfused with 4% paraformaldehyde, then post-fixed in the same for another 2 hours. There is some concern of tissues being over-fixed with excessive cross-linking that would impair Chx10 staining. This would be addressed with inclusion of anatomy.

- Figure 3A: why are there 3 traces aligned horizontally, what are the 3 different traces? If different examples and/or timepoints, maybe indicate.

- Figure 4C: why is the peak RMS not at zero when there are “no bouts”? How are bouts defined?

- p23, discussion “Importantly, the requirement for V2a neuron activity for breathing in neonatal mice indicates that dysfunction of these neurons could contribute to sudden infant death syndrome (SIDS) in humans”. This comes a bit out of the blue, what is the evidence that irregularity increases the risk of SIDS?

- Discussion p25 : “Thus, dual roles for V2a neurons in either activating (Romer et al. 2017) or inhibiting (this study) ARM activity is consistent with their dual roles in locomotor circuits.” This is not clear, what is meant by dual role? In the locomotor circuit, I seem to understand that the 2 cited papers have interfered with 2 different populations (spinal or brainstem). Here, there is no information about the location of the cells. In addition, the outcome of activating or silencing is largely similar (my major concern 1), so I am not sure that dual is appropriate here.

## Author Response

The authors thank the reviewers for their insightful comments and suggestions. The manuscript has been revised to address their concerns as detailed below. The modified portions of the manuscript are highlighted.

1) The authors describe in detail the anatomical studies they performed, and the quantitative analysis made on the collected tissues. However, without any figures of this anatomy, it is very hard to put their results into context. Show the HA-tagged and the Chx10 labeled cells in the neonatal and adult tissue collected from both medulla and spinal cord. It will be important to see the cells described and quantified, and possibly their brainstem and spinal distributions if the tissue retained allows it. This will also better highlight differences between the 2 anatomical locations in the two developmental stages studied.

Could the authors please also clarify why only spinal cord sections from C4 were used for quantification (only even then only every 8th section), when spinal cord from C1 to C6 was sectioned. Was any software used to assist with analysis?

We now provide a new Figure 1 that shows the HA-Tag (DREADD) and Chx10 expressing cells in the medulla and spinal cord from both neonatal (P2) and adult mice. Quantitation of HA-Tag+/Chx10+ cells is now reported for neonatal V2a-(Gi)DREADD mice in paragraph 1 of the results section (pages 14-15) along with the adult counts.

Since the number of V2a (and other neurons) vary along the rostro-caudal axis, we counted cells at C4 in all cords to eliminate potential differences that could arise from counting cells at different axial levels. Although any of the segments could have been selected as a standard reference point, we selected C4 because it is easily identified based on the distinct morphology of the phrenic nucleus in this segment. This point is now clarified in the methods section. We counted cells from at least 9 hemisections per animal for each age and tissue type. We now provide additional details on the counting of neurons in neonatal and adult mice including our use of NIS Elements software to perform counting (pages 6-7).

2) The authors clearly document functional effects of silencing those cells expressing the DREADD receptor, but an important point to note is that neurons in both the medulla and spinal cord are affected by CNO delivery, and these are likely two quite different populations of neurons. This needs to be considered when then drawing conclusions about the contribution these cells make respiratory activity. The authors need to either i) address this with targeted silencing of one population over the other, or ii) carefully discuss their data, limitations and interpretations in light of this fact.

The discussion has been expanded to address the limitation that the V2a-(Gi)DREADD mouse line silences both brainstem and spinal cord V2a neurons (pages 31-32). Additionally, the authors address the different roles that different subpopulations of V2a neurons may have on respiration (Page 30) and clarify that future studies are needed to address how brainstem vs. spinal V2a neurons control respiratory rhythm and pattern (Page 31).

3) In the adult, the increase of respiratory frequency and mobilization of accessory respiratory muscles bouts following V2a silencing is strikingly reminiscent of another study: if I understood right, Romer et al. (2017) reported very similar findings by activating the same neurons (e.g. compare current fig 4 with Fig 4 or Romer et al). How can an opposite interference of the same neurons' activity yield similar effects? The absence of mirror effects opens the possibility that one strategy (silencing or activating) is failing, which authors need to rule out. Also, the paper currently does not help to understand whether a possible strategy for enhancing respiratory function in ALS would be to favor, or on the contrary to inhibit, V2a neuronal activity.

a. Isn't it possible that over-activation may lead to inability to maintain spiking, or to change of the firing pattern, altogether leading to a functional inhibition? Authors should do the activation experiments themselves to verify the findings of the Romer paper in their own conditions, with appropriate CNO doses.

We are authors on the previous study in which the excitability of V2a neurons is increased, so we have extensively compared the two models directly. We rule out the possibility that one strategy (silencing or activating) is failing by showing different effects of CNO on diaphragm function and hindlimb reflexes in V2a-(Gi)DREADD mice versus V2a-(Gq)DREADD (referred to as “V2a-CHRM3” in Romer et al., 2017) mice. A new results section, “Increasing and decreasing the excitability of V2a neurons differentially affects motor functions” (pages 23-24) and accompanying Figure 7 was added along with discussion of these data on page 28.

In both the excitatory and inhibitory DREADD mouse lines, we tested and report the effect of a wide range of CNO doses on ARM activation (0.3 - 1.0 mg/kg*bw and 1.0 - 15.0 mg/kg*bw, respectively) (reported in this manuscript and in Romer et al. 2017). We see activation of ARMs at all doses and, as expected, higher frequencies at higher doses. Thus, we propose that EITHER activating or inhibiting V2a neurons could be potential therapies to improve ARM activity and enhance respiration. The discussion now includes this assertion as well as considerations for evaluating which may be the best approach (Page 32).

b. Alternatively, the timing might be important; however here there are no time-points following CNO administration and the timing used is not clearly stated. It seems that recordings are performed immediately after CNO administration, for 1h, is that right? However, several groups have reported peak effects at later time-points (between 1h and 2h). Can authors be more specific regarding time-points and check or report the effects over a longer period of time?

We have modified the figure (now Figure 4E) to include trapezius bout frequency plots for each mouse over the course of the hour long CNO treatment so the timing of ARM activation is clearly reported. The x-axis shows the length of the recording following CNO administration from 0 minutes to 60 minutes (end of the recording). The time periods where each mouse is at rest is indicated by grey bars to clearly identify the periods included in the analysis of ARM activity. These results are explained on page 17 in the results section.

c. Finally, it could be expected that V2a inhibited mice have a dis-regulated baseline locomotor activity (Bretzner 2013; Bouvier 2015), with a possible indirect impact on ARM. Have authors controlled for this?

Although silencing V2a neurons in the rGi reduces the ability to halt ongoing locomotion, this did not impact our studies. We only examined respiratory muscle activity in a plethysmograph chamber when the animals were at rest and not during movement, much less open field running. However, we did assess the amount of time each animal spent at rest in the plethysmography chamber before and after CNO administration and found that it did not change (47.3 {plus minus} 5.2% vehicle vs. 50.7{plus minus}5.8% CNO , n=9, p=0.670) as we now report in the results section on Pages 18-19). This is not surprising since the confined space of the plethysmography chamber prevents animals from initiating locomotor behavior, and thus there is no opportunity to test the ability of animals to halt ongoing activity. We also now report the periods during which each animal is at rest in Figure 4E.

In any case, authors should also include a deep discussion paragraph on these discrepancies.

We now include an additional results section demonstrating that although both activation and inhibition of V2a neurons can increase ARM activity, we observe distinct effects on other motor behaviors (i.e. hindlimb extension reflex and diaphragm activity) when V2a neurons are silenced vs. activated in the V2a-(Gi)DREADD and V2a-(Gq)DREADD mouse lines, respectively. The discussion now addresses these results as well as proposes a hypothetical model (page 30) that is consistent with the results from both the V2a-(Gi)DREADD and V2a-(Gq)DREADD mouse lines. The model proposes that one subset of V2a neurons (that are normally silent at rest) participates in an excitatory pathway to activate V2a neurons when needed (i.e. locomotion). The model proposes that another subset of V2a neurons (active at rest) participates in an inhibitory pathway to prevent ARM activation when they are not needed. The discussion also includes an alternative hypothesis- compensation by ARM MNs (but not diaphragm MNs) after the loss of V2a input increases their excitability- as well as potential ways to distinguish between the two hypotheses in future experiments (pages 31-32).

4) Authors report that V2a inhibition increases breathing and diaphragm frequency by 15% (2C; 2E) but plots in 2G and 2H show a similar breathing frequency between the two groups throughout the recording. These plots of instantaneous frequency are not convincing (compare with the newborn situation, which is). In fact the value (~250 / min) is above average of the group data in 2C-F and seem to concern an animal that does not increase. Can authors verify their plots and/or bar-graphs and maybe select a trace that is a representative of the group data?

We thank the reviewers for pointing out that the previous frequency plot is not representative of the average group data shown in Figure 2 (which is now Figure 3). The plots in Figure 3G and 3H have been replaced with an animal's data set that is representative.

5) Introduction, last paragraph, page 5: “we uncovered an unexpected role for V2a neurons in suppressing extradiaphragmatic activity at rest (...) circuit that actively prevents activation of ARMs”. This is not shown in the work. This would imply that activating V2a during ARM bouts suppresses them; this is not done here. Please remove/rephrase.

This phrase has been changed to, “In addition, we show that silencing V2a neurons results in activation of ARMs in adult mice at rest, suggesting that these neurons play an important role in patterning extradiaphragmatic respiratory muscle activity.” (page 5).

Minor comments :

- Please correct spelling of “pre-Boetzinger” to “pre-Bötzinger”

This spelling has been corrected.

- The authors state that tissues were transcardially perfused with 4% paraformaldehyde, then post-fixed in the same for another 2 hours. There is some concern of tissues being over-fixed with excessive cross-linking that would impair Chx10 staining. This would be addressed with inclusion of anatomy.

Figure 1 now includes pictures of Chx10 and HA-Tag staining of the cervical spinal cord and brainstem at the level of the nucleus ambiguous in neonatal and adult mice. We used a standard fixation protocol that we and others have used for Chx10 staining in previous publications. However, we added additional details to the methods section (pages 6-7) to clarify our procedures and ensure that they can be faithfully replicated.

- Figure 3A: why are there 3 traces aligned horizontally, what are the 3 different traces? If different examples and/or timepoints, maybe indicate.

Figure 3A (Figure 4A in the revised manuscript) shows different examples of bouts from the same V2a-CHRM4 mouse following CNO administration. The figure legend has been modified to report this clearly. We chose to display multiple short time frames rather than one long time frame with multiple bouts in order to show more detailed EMG and RMS traces.

- Figure 4C: why is the peak RMS not at zero when there are “no bouts”? How are bouts defined?

Based on Jensen et al., 2017, bouts are defined by three criteria that are reported in the “EMG Analysis” methods section, which we have now edited for clarity. EMG activity is scored as a “bout” if it satisfies the following 3 criteria:

1) the RMS EMG trace must exhibit at least 3 consecutive points that are at least 50% higher than the surrounding baseline (each point represents a 30 ms window).

2) EMG activity occurs during resting breaths (i.e. i.e. sighs and movement artifacts are excluded)

3) EMG activity occurs when the mouse is still (i.e. no movement or changes in posture) based on the synchronized video recordings.

The peak RMS is normalized to activity during a sigh (near maximal activity) per Mantilla, et al. 2011 to reduce intra-animal variability over time as opposed to normalizing to minimal EMG activity to reduce the baseline to zero. Thus, the average peak RMS during baseline activity reflects the average background EMG activity. We have added a description of the normalization procedure to the methods section rather than relying solely on our previously published methods (Jensen et al., 2017).

- p23, discussion “Importantly, the requirement for V2a neuron activity for breathing in neonatal mice indicates that dysfunction of these neurons could contribute to sudden infant death syndrome (SIDS) in humans”. This comes a bit out of the blue, what is the evidence that irregularity increases the risk of SIDS?

We have removed this speculative statement.

Discussion p25 : “Thus, dual roles for V2a neurons in either activating (Romer et al. 2017) or inhibiting (this study) ARM activity is consistent with their dual roles in locomotor circuits.” This is not clear, what is meant by dual role? In the locomotor circuit, I seem to understand that the 2 cited papers have interfered with 2 different populations (spinal or brainstem). Here, there is no information about the location of the cells. In addition, the outcome of activating or silencing is largely similar (my major concern 1), so I am not sure that dual is appropriate here.

We regret that our discussion of this topic was not clear and we have extensively revised this section of the discussion. In particular, our use of the word “dual” was misleading and implied that one neuron performed multiple roles, rather than distinct sets of V2a neurons playing different roles. The revisions include changing the word from “dual” to “different” when describing different roles of distinct subsets of V2a neurons. In addition, we more fully describe the experiments investigating the role(s) of V2a neurons in reticulospinal activation of locomotor circuits, including the locations of the cells being tested. Together, these studies provide evidence that distinct sets of V2a neurons may be involved in initiating and halting locomotion, which many would consider “opposing” functions. We see this as analogous to our newly proposed model whereby distinct subsets of V2a neurons may promote and inhibit ARM activity. Moreover, the discussion has been expanded to include an additional example of two distinct subsets of V2a neurons within the lumbar spinal cord that play different roles in locomotion (Shox2+/Chx10+ vs. Shox2-/Chx10+) where one appears to functionally activate motor neurons and the other inhibits motor neurons (on the opposite side of the cord). We acknowledge in the discussion that future experiments targeting subpopulations of V2a neurons are warranted to resolve the roles of different V2a subtypes in different regions of the CNS or even within the same segment of the spinal cord.
